# Effects of the Deletion of Early Region 4 (*E4*) Open Reading Frame 1 (*orf1*), *orf1-2*, *orf1-3* and *orf1-4* on Virus-Host Cell Interaction, Transgene Expression, and Immunogenicity of Replicating Adenovirus HIV Vaccine Vectors

**DOI:** 10.1371/journal.pone.0076344

**Published:** 2013-10-15

**Authors:** Michael A. Thomas, Rui Song, Thorsten Demberg, Diego A. Vargas-Inchaustegui, David Venzon, Marjorie Robert-Guroff

**Affiliations:** 1 Section on Immune Biology of Retroviral Infection, Vaccine Branch, National Cancer Institute, National Institutes of Health, Bethesda, Maryland, United States of America; 2 Biostatistics and Data Management Section, National Cancer Institute, National Institutes of Health, Bethesda, Maryland, United States of America; Boston College, United States of America

## Abstract

The global health burden engendered by human immunodeficiency virus (HIV)-induced acquired immunodeficiency syndrome (AIDS) is a sobering reminder of the pressing need for a preventative vaccine. In non-human primate models replicating adenovirus (Ad)-HIV/SIV recombinant vaccine vectors have been shown to stimulate potent immune responses culminating in protection against challenge exposures. Nonetheless, an increase in the transgene carrying capacity of these Ad vectors, currently limited to approximately 3000 base pairs, would greatly enhance their utility. Using a replicating, *E3*-deleted Ad type 5 host range mutant (Ad5 hr) encoding full-length single-chain HIV_BaL_gp120 linked to the D1 and D2 domains of rhesus macaque CD4 (rhFLSC) we systematically deleted the genes encoding early region 4 open reading frame 1 (*E4orf1*) through *E4orf4*. All the Ad-rhFLSC vectors produced similar levels of viral progeny. Cell cycle analysis of infected human and monkey cells revealed no differences in virus-host interaction. The parental and *E4*-deleted viruses expressed comparable levels of the transgene with kinetics similar to Ad late proteins. Similar levels of cellular immune responses and transgene-specific antibodies were elicited in vaccinated mice. However, differences in recognition of Ad proteins and induced antibody subtypes were observed, suggesting that the *E4* gene products might modulate antibody responses by as yet unknown mechanisms. In short, we have improved the transgene carrying capacity by one thousand base pairs while preserving the replicability, levels of transgene expression, and immunogenicity critical to these vaccine vectors. This additional space allows for flexibility in vaccine design that could not be obtained with the current vector and as such should facilitate the goal of improving vaccine efficacy. To the best of our knowledge, this is the first report describing the effects of these *E4* deletions on transgene expression and immunogenicity in a replicating Ad vector.

## Introduction

Vaccines are essential tools in the global effort to reduce deaths due to multiple diseases. To date, successful vaccines have been developed against the proverbial “low hanging fruit;” however, the continued lack of effective vaccines against diseases such as malaria, tuberculosis, and HIV/AIDS underscores the need for even greater efforts aimed at the design and development of preventative vaccines. The recent body of literature on replicating adenovirus (Ad) is replete with evidence of its promising use as a vaccine delivery vector. As part of a preventative HIV vaccine strategy, it has been shown to elicit potent humoral and cellular immune responses [1]. Most importantly for HIV, the replicating Ad vaccine vector targets and persists at mucosal sites [2] where HIV makes its initial entry [3]. In combination with envelope protein boosts, immunization with replicating Ad-HIV/SIV recombinants has elicited strong protection against HIV, SIV, and simian/human immunodeficiency virus (SHIV) challenges in rhesus macaque and chimpanzee models [4]–[9]. When compared to a replication-deficient early region 1 and 3–deleted Ad (Ad5*ΔE1ΔE*3), replicating Ad induced better transgene immune responses at the same or a lower dose [10]. Today, replicating Ad sub-type 4 (Ad4ΔE3) is being developed as a delivery vector for both HIV/AIDS and influenza vaccines [11], [12].

The *E3* region of Ad is dispensable for virus replication [13], [14] and is deleted from most Ad vaccine vectors. With the added deletion of the *E1* region, the transgene carrying capacity of the first generation Ad5*ΔE1ΔE3* vaccine vectors is about five thousand base pairs (5 kb). The replicating Ad vector with only deletion of the nonessential *E3* region is restricted to carrying transgenes of about 3 kb in size [15]. This limited transgene capacity undermines the clinical potential of the replicating vector. To address this limitation we took advantage of the fact that Ad5 with deletions of *E4orf1* through *E4orf4* produce viral progeny, synthesize viral DNA, and induce the production of late viral proteins comparably to the wild-type virus [16], [17].

The *E4orf1* gene product negatively regulates late viral protein synthesis and levels of viral progeny produced, and also promotes survival in Ad5-infected cells [18]. No specific role or function has been ascribed to the product of the *E4orf2* gene. The functions of the E4orf3-encoded protein include aiding in the shut-off of cellular protein synthesis and enhancing nuclear export of viral mRNA. Additionally, it suppresses viral induced DNA damage in a manner involving the sumoylation of sequestered Mre11 and Nbs1 [19]. E4orf3 also inactivates the interferon induced cellular antiviral defense mechanism by mislocalizing Daxx, sp100, and PML [20]. In an *E1B55K*-deleted virus, *E4orf3* may be required both for late viral protein synthesis and viral progeny production [21]. Interestingly, of the *E4* gene products, E4orf3 is the only one shown to enhance the longevity of transgene expression from a CMV promoter in an *E1*-deleted vaccine vector [22]. Most of the activities of the *E4orf4* gene product may be accounted for by its interaction with protein phosphatase 2A (PP2A). These include the hypophosphorylation of various viral and cellular proteins, facilitating alternative splicing of Ad mRNAs [23], and regulating protein translation through an interaction with the mammalian target of rapamycin (mTOR) pathway [24]. E4orf4 also represses the *E2* region [25] and thus may regulate levels of viral DNA accumulation. In spite of these varied functions, viruses lacking these *E4* gene products remain phenotypically wild-type suggesting the expressed proteins are not needed for a productive infection.

Because *E4orf1* through *E4orf4*-deleted viruses created previously [16], [17] did not contain a transgene, the effect of such deletions on transgene expression and immunogenicity had not been evaluated. Therefore, we created a replication-competent *E3*-deleted Ad type 5 host-range mutant (Ad5 hr)-recombinant encoding full-length single chain HIV_BaL_gp120 attached to a flexible linker and the first two domains of rhesus CD4 (rhFLSC) [26], and systematically deleted *E4orf1*, *1–2*, *1–3*, and *1–4*. The results of this study provide evidence that while deletion of *E4orf1* through *E4orf4* expands the transgene carrying capacity of replicating Ad vectors, these deletions have little to no effect on virus-host cell interaction, transgene expression, T-cell immunogenicity, or transgene-specific antibody binding titers. Surprisingly, sera from mice vaccinated with the *E4*-deletion variants showed differential binding to Ad antigens, suggesting that the *E4* gene products may harbor some yet to be uncovered functions that may modulate antibody responses.

## Results

### Construction of MAd5rhFLSC variants containing deletions of *E4orf1*, *1–2*, *1–3*, and *1–4*

**Figure 1:**
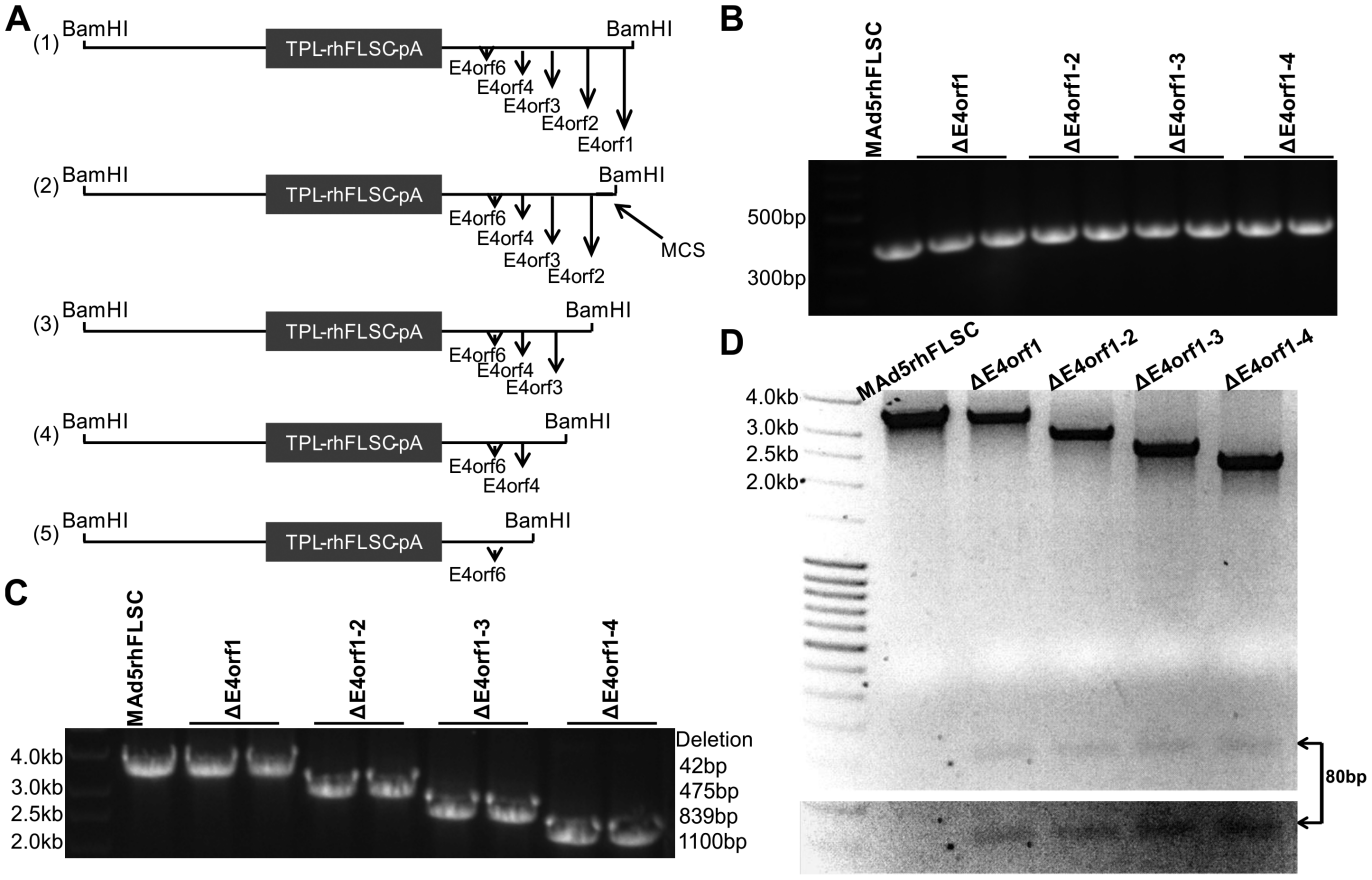
Construction and verification of Ad5-rhFLSC variants with deletions in *E4orf1*, *1–2*, *1–3*, and *1–4*. (A) Schematic diagram of gel purified BamHI digested pBRAd5ΔE3 (TPL-rhFLSC-pA) shuttle plasmids. (**B**) Using the primers listed in Table S1 PCR products were obtained from the parental Mad5rhFLSC and deleted viruses and run on a 0.8% agarose gel. The fragment from the ΔE4orf1–4 virus containing an 1100 bp deletion, the ΔE4orf1–3 virus containing an 839 bp deletion, the ΔE4orf1–2 virus containing a 475 bp deletion and the ΔE4orf1 virus containing a 42 bp deletion were run with Mad5rhFLSC as a control. (**C**) NsiI digestion liberated an 80 bp fragment seen at the bottom of the gel in each of the E4-deleted viruses but not in the parental virus MAd5rhFLSC. The bottom portion of the gel was overexposed to provide better visualization of the 80 bp fragment. (**D**) The concentrations of the purified viruses were obtained by optical density (1 OD unit at 260 = 1.0×10^10^ PFU/mL) and plaque assays on 293 cells. The particle to plaque ratios are shown.

Ad recombinants were generated as described in Materials and Methods, and outlined in Figure 1A. Positive clones were evaluated for appropriate deletions in the *E4* region (Figure 1B). The PCR fragment from the *ΔE4orf1–4* virus (a deletion of about 1103 bp) migrated the fastest followed by that of the *ΔE4orf1–3* virus (a deletion of about 839 bp), and that of the *ΔE4orf1–2* virus (a deletion of about 475 bp). The presence of the inserted rhFLSC gene in plaque-purified isolates was confirmed by PCR (data not shown). The Δ*E4orf1* virus (a deletion of 42 bp) remained indistinguishable from the parent virus. To address this, we isolated the PCR product from each of the viruses and digested it with the NsiI restriction enzyme. This liberated an 80 bp fragment seen at the bottom of the gel (Figure 1C). This fragment is present in the lanes of all *E4*-deleted viruses but not in the parental control lane. This result not only serves to distinguish the Δ*E4orf1* virus from the parent but also confirms the presence of the unique multiple cloning sites (MCS) in all the *E4*-deleted viruses. The virus concentrations and particle to plaque ratios are shown in Figure 1D.

### Deletion of *E4orf1* through *E4orf4* has little effect on viral progeny production

**Figure 2:**
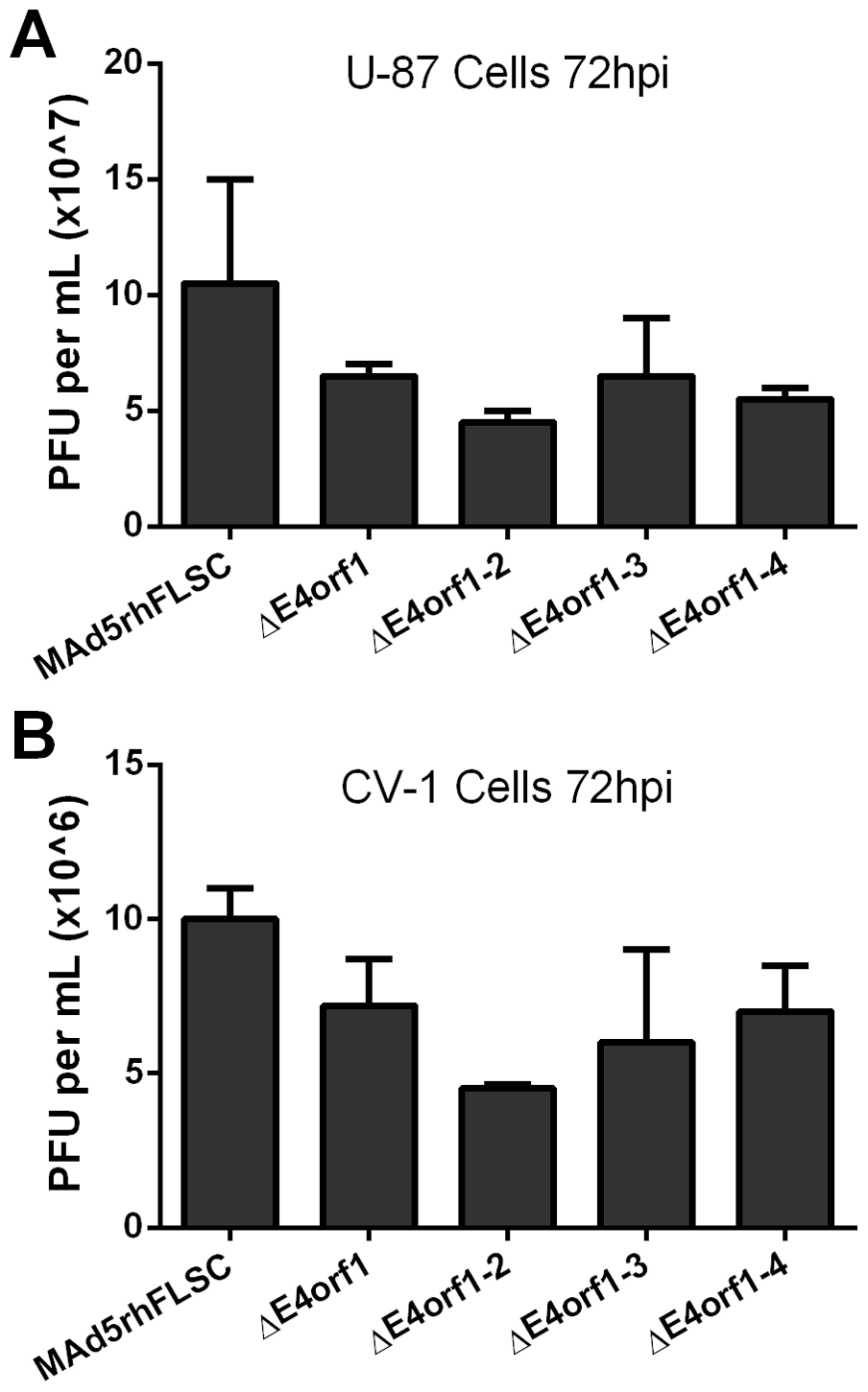
Deletion of *E4orf1* through *E4orf4* has little effect on viral progeny production. (**A**) U-87 cells were infected with the listed viruses at an MOI of 5 PFU/cell for 72 hours. The cells were collected and after three rounds of freezing-thawing the media were used to infect 293 cells as described in Materials and Methods. The plaques were counted and numbers graphed and analyzed. No significant differences between the parental and *E4*-deleted viruses were obtained using one-way ANOVA (p>0.05). The values shown are the means of 2 independent infections ± SEM. (**B**) The results were confirmed in CV-1 cells (p>0.05).

To assess effects of the deletions on virus progeny production, we infected U-87 (Figure 2A) and CV-1 (Figure 2B) cells and performed plaque assays as described elsewhere [18]. CV-1 cells were included as the viruses used here were constructed in the Ad5 hr vector in which a single amino acid substitution in the DNA binding protein allows replication in monkey cells [27]. Thus our vectors can be used in pre-clinical vaccine studies in non-human primates. No significant differences were observed among the viruses. These results support those obtained by Huang and Hearing [16] and extend them to *E3*-deleted transgene bearing vectors. We conclude that the combined deletions of *E3* and *E4orf1* to *E4orf4* have little effect on the replication potential of these vaccine vectors.

### The virus host-cell interaction is unchanged by the deletion of *E4orf1* through *E4orf4*

Upon binding to its host-cell Ad enters via receptor-mediated endocytosis. The viral particle then travels along intracellular filaments, attaches to the nucleus, and there deposits its DNA. Once in the nucleus a pattern of temporal viral gene expression ensues, beginning with expression of the immediate early gene, *E1A*, followed by the other early genes, *E1B*, *E2A*, *E2B*, *E3* and *E4*. Ad quickly commandeers the cellular machinery that controls cell cycle progression [28], [29] primarily by the action of E1A on the retinoblastoma protein pRb. E1A binding to pRb results in the release and activation of the E2F transcription factor [30]. Activated E2F upregulates many genes that are required for S- and the other phases of the cell cycle. In conjunction with its role in the activation of E2F, E1A also targets the dual-specificity protein phosphatase cell division cycle 25 A (Cdc25A) [31], which regulates cell cycle progression by removing the inhibitory phosphorylation on cyclin-dependent kinases [32]. By these coordinated actions E1A drives cell cycle progression in infected cells.

**Figure 3:**
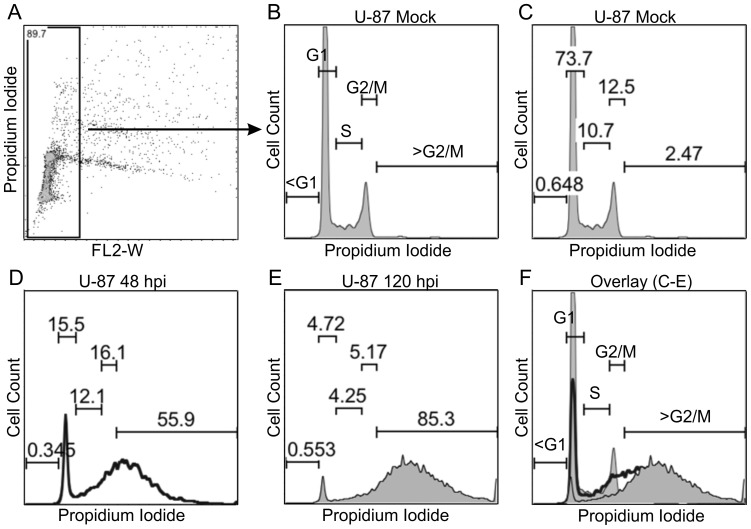
Quantification of cell cycle phases. (**A–F**) Cells were treated and cycle phases analyzed as described in Materials and Methods. (**A**) All the cells were gated to reduce doublets by selection of cells within the inset rectangular box. (**B**) Mock-infected cells in the cell cycle phases G1, S, G2/M, <G1, and >G2/M are depicted. (**C**) Proportion of mock-infected cells in each phase of the cell cycle. (**D, E**) Representative plots and percentages of U-87 cells 48 (**D**) and 120 (E) hpi. (**F**) An overlay of plots C–E.

To determine whether deleting *E4orf1* to *E4orf4* altered the ability of the virus to regulate cell cycle progression, we interrogated the DNA cell cycle profile of asynchronously growing HeLa, U-87, and CV-1 cells. The cells were gated to reduce doublets (Figure 3A) and each cell cycle phase defined (Figure 3B). Cells in the G1 phase of the cell cycle constitute the majority of cells in culture, and thus the counts are highest for that population. The population labeled G2/M contains about two times the DNA content of the G1 cells. Between G1 and G2/M are S-phase cells that are synthesizing DNA, and contain an intermediate DNA content. Dead or dying cells contain fragmented DNA and are labeled <G1. In some instances cells by-pass the normal somatic cell cycle and accumulate more DNA than G2/M cells. This altered cell cycle phase is termed endoreduplication and is labeled here as >G2/M. Representative examples of mock and Ad-infected U-87 cells are depicted (Figure 3C-E) with an overlay of plots C-E shown in Figure 3F.

**Figure 4:**
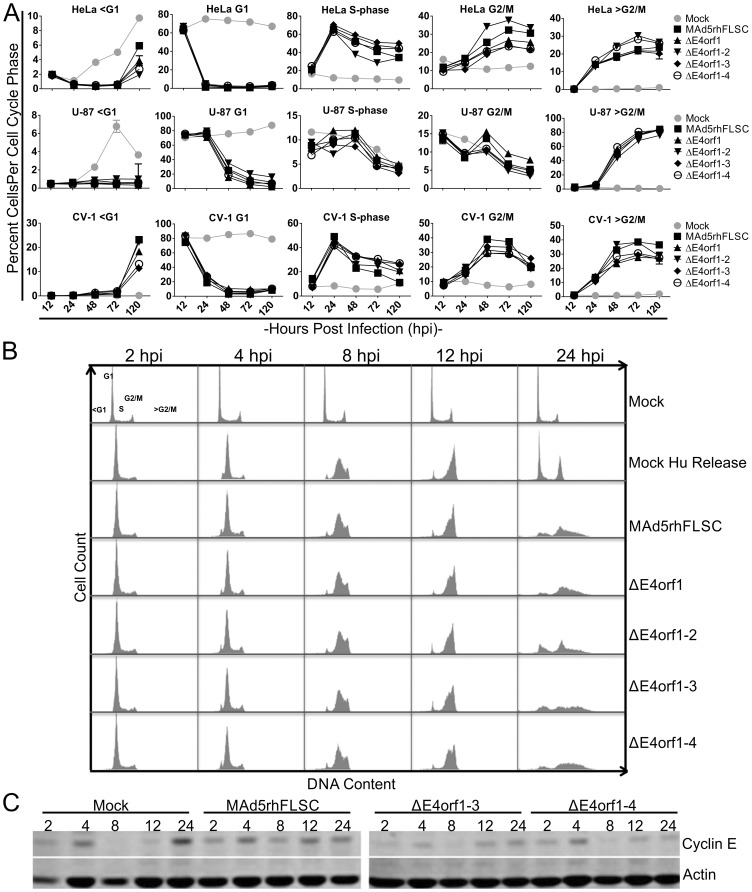
Deletion of *E4orf1* through *E4orf4* does not affect Ad's regulation of the cell cycle. (**A**) HeLa, U-87, and CV-1 cells were mock-infected or infected with the parental or E4-deletion variants for the indicated periods of time. The cells were subsequently processed for cell cycle analysis. The DNA profiles of all cells infected with the *E4*-deleted viruses were similar to those of cells infected with the parental virus in all cells at all time points. The values shown are the means of 5 independent infections per time point per cell line, ± SEM. (**B**) HeLa cells were exposed to 2 mM hydroxyurea (HU) overnight. The cells were released from the HU block and exposed to media alone (HU Release) or virus containing media as shown. The cells were collected at the indicated times and processed for cell cycle analysis. Mock infected cells not exposed to HU are shown in the top row. (**C**) Cells were lysed directly in boiling 1X protein sample buffer and the total cell lysates were analyzed by western blot using anti-cyclin E and anti-actin antibodies. Figures are representative of 2–3 independent experiments.

In each of the three interrogated cell lines, the G1 population of the mock-infected cells varied only slightly over time. This was in stark contrast to the G1 populations of the infected cells (Figure 4A column 2). The S-phase and G2/M infected cell populations initially increased but thereafter decreased over time (Figure 4A columns 3 and 4). These two populations varied in the mock-infected cells. Interestingly, a significant fraction of the infected cells had DNA >G2/M that increased over time (Figure 4A column 5). The fractions of *E4*-deleted infected cells undergoing endoreduplication were similar to those of cells infected with the parental virus. Thus deletion of *E4orf1* through *E4orf4* did not alter Ad-induced cell cycle progression or levels of endoreduplication in asynchronously growing cells.

To confirm our findings and further assess the effects of the *E4* deletions on cell cycle progression, we evaluated the DNA profile of hydroxyurea (HU) synchronized infected HeLa cells. HU arrests cells at the G1/S border because it inhibits ribonucleotide reductase, thus reducing the pool of available deoxyribonucleotides needed for cells to complete S-phase. After being released from HU for two hours, the majority of the mock-infected cells were in late G1 to early S-phase (Figure 4B, row 2, column 1). Four hours later they were in early to mid S-phase (Figure 4B, row 2, column 2). Eight hours later, the majority of the mock-infected cells were in late S-phase (Figure 4B row 2 column 3) and by 12 hours post-HU release they were mainly in G2/M (Figure 4B, row 2, column 4). By 24 hours post release, the mock-infected cells had for the most part regained a near normal cell cycle profile (Figure 4B, row 2, column 5). The DNA profiles of virus- and mock-infected HU-release cells were indistinguishable up until 24 hpi (Figure 4B, row 2–6, column 1–4). At 24 hours only the virus-infected cells had DNA >G2/M (Figure 4B, row 3–6, column 5). These results confirmed our findings shown in Figure 4A, and extended them to synchronously growing cells.

It has been suggested that cyclin E is stabilized in Ad-infected cells [33]. In normal cells, cyclin E protein levels oscillate during the cell cycle, peaking during mid S-phase and falling soon after [34]. We monitored the levels of cyclin E in synchronized HeLa cells by western blot. In mock-infected cells, cyclin E increased 4 and 24 hours post HU release, with intervening decreases at 8 and 12 hours (Figure 4C, row 1, columns 1–5). By contrast, oscillation in cyclin E levels was diminished in virus-infected cells. Following an initial increase at 4 hrs post-HU release, a transient decrease was seen at 8 hrs. However, at 12 hrs and thereafter, cyclin E levels appeared relatively constant (Figure 4C row 1, columns 9–10, 14–15 and 19–20). These results support the findings of Zheng et al. [33], suggesting that cyclin E stabilization is a feature common to the pathogenesis of replicating Ad that occurs as shown here, independent of *E4orf1* to *4*. Taken together, deletion of *E4orf1* to *E4orf4* does not change the vector's ability to regulate cell cycle progression in infected cells.

### Transgene expression is unchanged by the deletion of *E4orf1* through *E4orf4*

**Figure 5:**
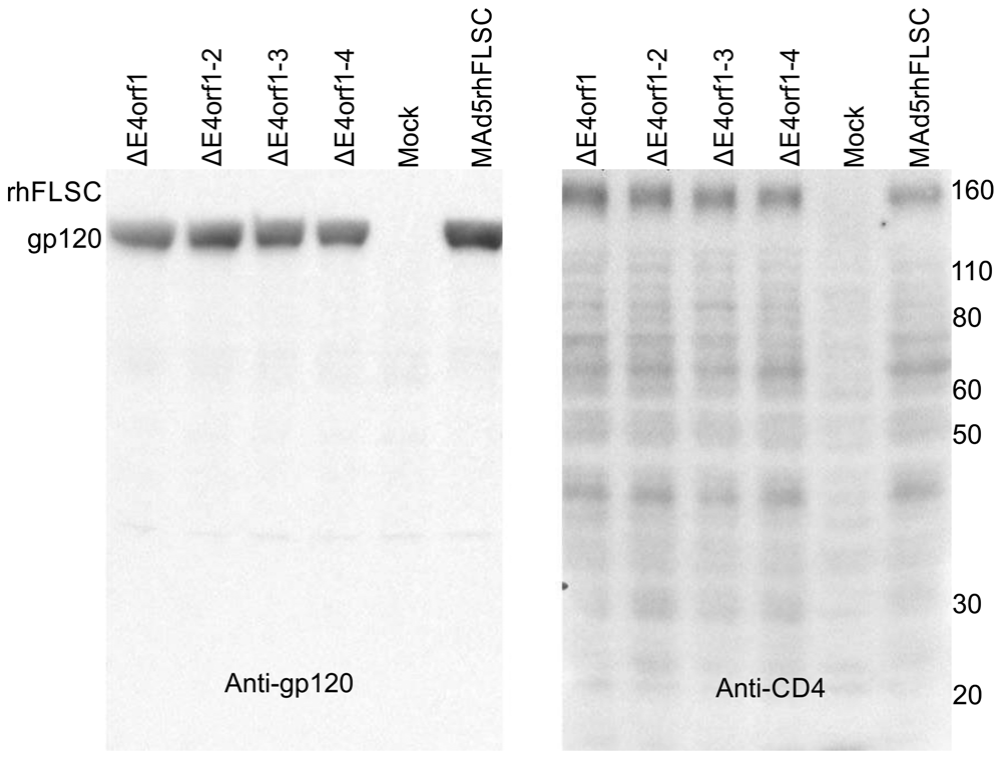
Deletion of *E4orf1* through *E4orf4* has no effect on transgene expression. HeLa cells were plated and the day after infected at an MOI of 50 PFU/cell with the indicated viruses. 48 hours post infection the cells were lysed directly in boiling 1X protein sample buffer and the total cell lysates were analyzed by western blot using either anti-gp120 or anti-CD4 to identify the rhFLSC protein. The figures shown are representative of 2–4 experiments.

To determine the effects of the deletions on transgene expression, we infected HeLa cells and monitored rhFLSC expression by western blot. The original FLSC molecule was characterized using anti-CD4 which recognized the intact molecule and a mixture of murine anti-gp120 monoclonal antibodies which recognized the intact molecule as well as the gp120-processed product [26]. Here our anti-gp120 monoclonal recognized an epitope only accessible on the processed gp120, and anti-CD4 was used to visualize the complete rhFLSC. As shown in Figure 5, the rhFLSC transgene was comparably expressed regardless of the *E4*-deletions. To our knowledge, this represents the first report showing the expression of a transgene from replicating *E3*-deleted Ad vectors deleted of *E4orf1* through *E4orf4*.

**Figure 6:**
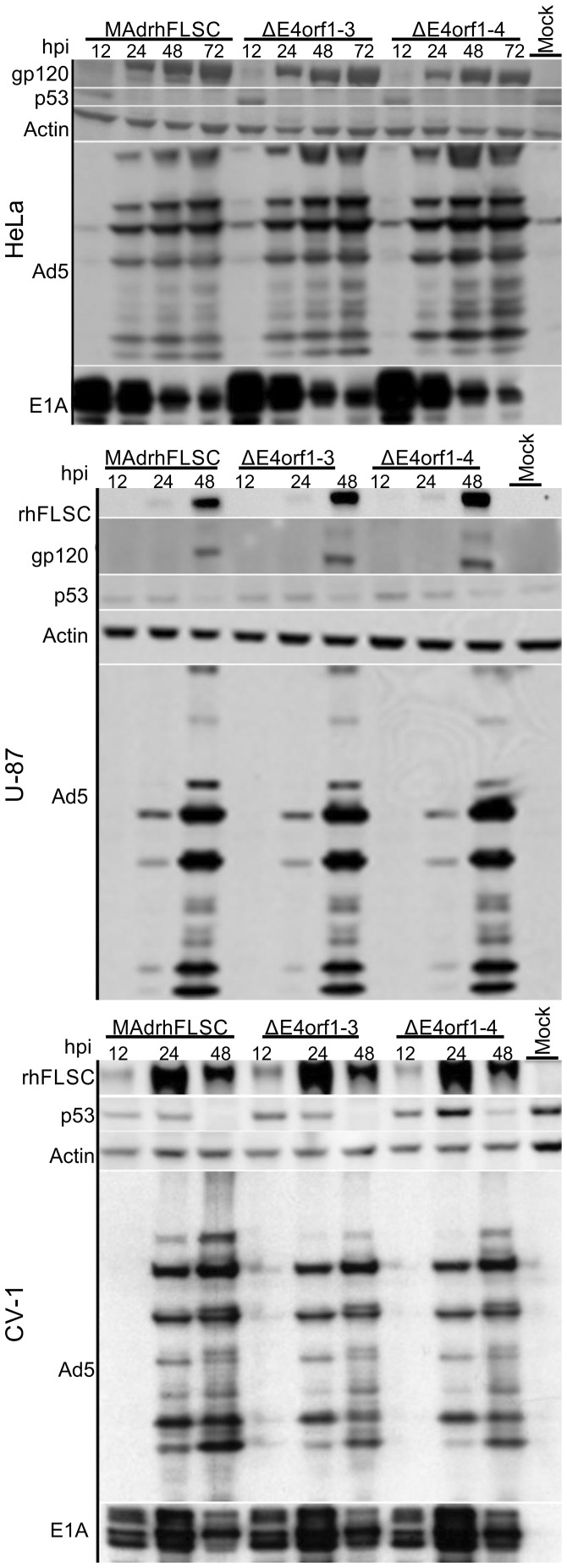
The transgene accumulates during the late phase of the virus infection cycle. HeLa, U-87, and CV-1 cells were plated and the day after infected at an MOI of 5–50 PFU/cell with the indicated viruses. At various times post infection the cells were lysed directly in 1X boiling protein sample buffer and the total cell lysates were analyzed by western blot using either anti-gp120, anti-CD4, anti-p53, anti-actin, anti-Ad5 or anti-E1A as indicated. Composite pictures showing results with the various antibodies are shown. The figures shown are representative of 2–4 independent infections.

The Ad replication cycle can be divided into an early and late phase with respect to the time at which the virus initiates replication of its DNA. In the late phase of the replication cycle, the structural genes, *L1*, *L2*, *L3*, *L4* and *L5* are expressed. Ad late genes are selected and expressed at the expense of cellular genes. Thus the time of the transgene accumulation may hint at how it is regulated. To assess this, we infected HeLa, U–87 and CV-1 cells with parental, *ΔE4orf1–3*, and *ΔE4orf1–4* viruses and evaluated the time of transgene expression. In general the transgene accumulated between 24-48 hpi like late viral proteins (Figure 6). The kinetics were similar to those reported for Ad7 encoding gp160 expressed under the control of the Ad major late promoter [35]. The temporal relationship observed suggests that the transgene is regulated in a manner similar to the late viral proteins.

Of the Ad *E4* gene products, E4orf6 is the only one known to affect levels of late viral proteins [16]. Since the levels of late viral proteins were unchanged by the deletions we made, it was reasonable to assume that the deletions did not interfere with the functionality of the E4orf6 gene product. To confirm this we took advantage of the fact that in wild type Ad infection an activity of the E1B55K/E4orf6 complex programs the tumor suppressor protein p53 for degradation [36]. Evidence of this activity is seen by the diminution of p53 expression over time in each of the cell lines evaluated (Figure 6). Taken together, these results along with Figures 2–4 suggests that the viruses we created phenotypically resemble wild-type viruses. Consequently, in an *E3*-deleted replication-competent Ad, further deletion of *E4orf1* through *E4orf4* leaves levels of late viral protein and transgene expression unchanged.

### Env- and Ad-specific memory T-cell responses are unchanged by the deletion of *E4orf1* through *E4orf4*

**Figure 7:**
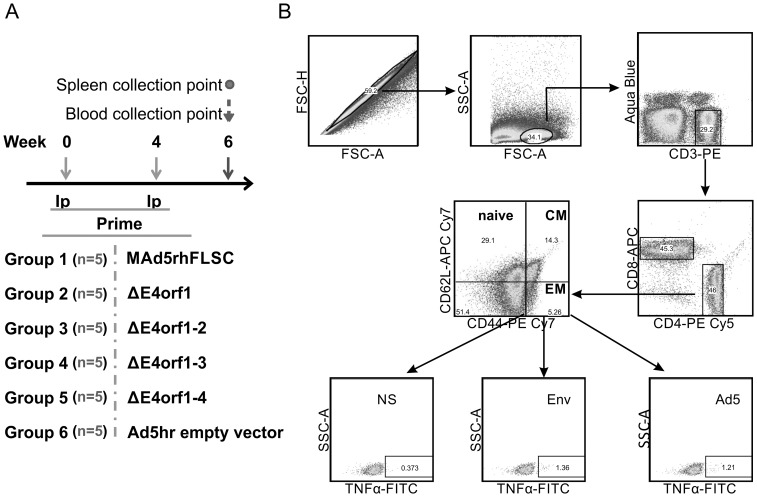
Vaccination Scheme and gating strategy. (**A**) Five 6–8 weeks of age female Balb/c mice per group were vaccinated at the indicated times with 5.0×10^8^ PFU of the listed viruses. On week six spleen and blood were collected from each animal. (**B**) Cells were first gated for singlets (Forward Scatter-High (FSC-H)/Forward Scatter-Area (FSC-A), lymphocytes (Side Scatter-Area (SSC-A)/FSC-A) and viable CD3 cells (aqua blue/CD3). CD8 and CD4 subpopulations were identified and further separated into either central or effector memory (CM and EM respectively) subpopulations based on CD62L and CD44 as indicated. Representative examples of non-stimulated (NS), gp120 envelope peptide stimulated (Env) and Ad5 fiber peptide (Ad5) stimulated EM cells producing TNFα are shown.

**Figure 8:**
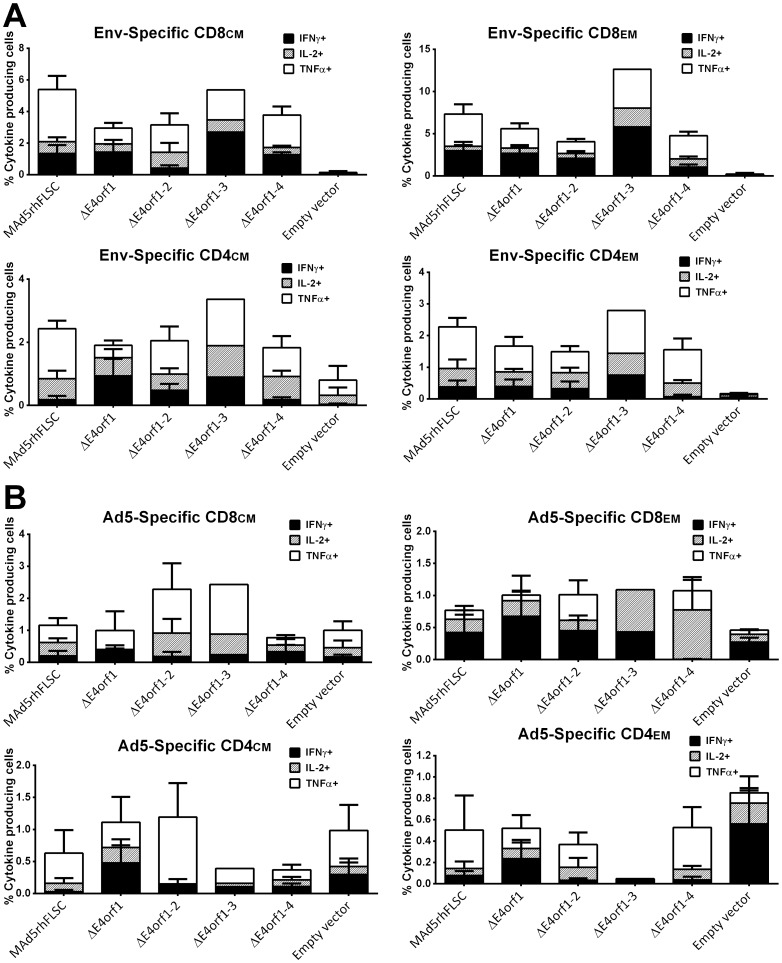
Deletion of *E4orf1* through *E4orf4* does not affect transgene or Ad5-specific cellular responses. (**A–B**) Intracellular cytokine staining of splenocytes with stacked responses for Env- or Ad5-specific CD8 and CD4 CM or EM T cells secreting IFNγ, IL-2, and TNFα. (A) No differences were observed between the parent and the *E4*-deleted viruses (p>0.05). Values of the five vaccinated groups were significantly different from those of the empty vector group for Env-specific CD8 CM and CD8 EM T cells (p<0.001 for both) and for Env-specific CD4 EM T cells (p<0.002) but not for Env-specific CD4 EM T cells (p>0.05). (B) No differences were observed between the parent and the E4-deleted viruses for the Ad5-specific memory responses (p>0.05). No differences were observed between vaccinated groups and the empty vector group (p>0.20). For ΔE4orf1-3 only cells from a single mouse were evaluated. For group 6 cells from 4 mice were evaluated. All the other experiments were performed using 5 mice. Stacked mean values of individual cytokines are plotted with error bars indicating SEM.

The replication of Ad5 is severely muted in mice – an effect of its host-range restriction. However, the viral genes, along with the transgene, are expressed in mouse cells allowing assessment of potential contributions of the *E4* gene products to transgene immunogenicity prior to more extensive evaluation of selected variants in a non-human primate model. Accordingly we evaluated T-cell immune responses by comparing levels of intracellular cytokines produced by splenocytes from groups of Balb/C vaccinated mice as outlined in Figure 7A and described in Materials and Methods. Figure 7B illustrates the gating strategy used to interrogate the T cell memory compartment. Env-specific IFNγ, IL-2, and TNFα positive cells were observed in all memory compartments of CD8 and CD4 cells (Figure 8A). Among CD8 cells, cytokine expression was significantly higher in all the immunized groups compared to the empty vector group (Figure 8A row 1), with the exception of the *ΔE4orf1–3* vaccinated animals where statistical analysis was not possible as only cells from one animal were viable due to suboptimal processing. Similar statistical significance was seen for CD4 effector memory (EM) cells, whereas among CD4 central memory (CM) cells, cytokine expression did not reach statistical significance (Figure 8A row 2). The percentage of cytokine-producing cells was comparable between groups vaccinated with the parental and *E4*-deleted viruses (Figure 8A). From this we conclude that deletion of *E4orf1* to *E4orf4* did not significantly alter virus-induced transgene-specific cellular responses in vaccinated animals.

We next analyzed Ad-specific T-cell immune responses following stimulation of splenocytes with Ad5 fiber peptides. The responses to Ad5 fiber were low in comparison to transgene-specific responses (Figure 8A, B). However, the percentages of cytokine producing cells in response to Ad fiber peptides were comparable across groups for both CD8 and CD4 central memory (CM) and effector memory (EM) cells (Figure 8B). Therefore, deletion of *E4orf1* through *E4orf4* has little to no effect on the vectors' ability to induce Ad-specific cellular responses in vaccinated animals.

### Transgene-specific antibody binding titers are unchanged by the deletion of *E4orf1* through *E4orf4*

**Figure 9:**
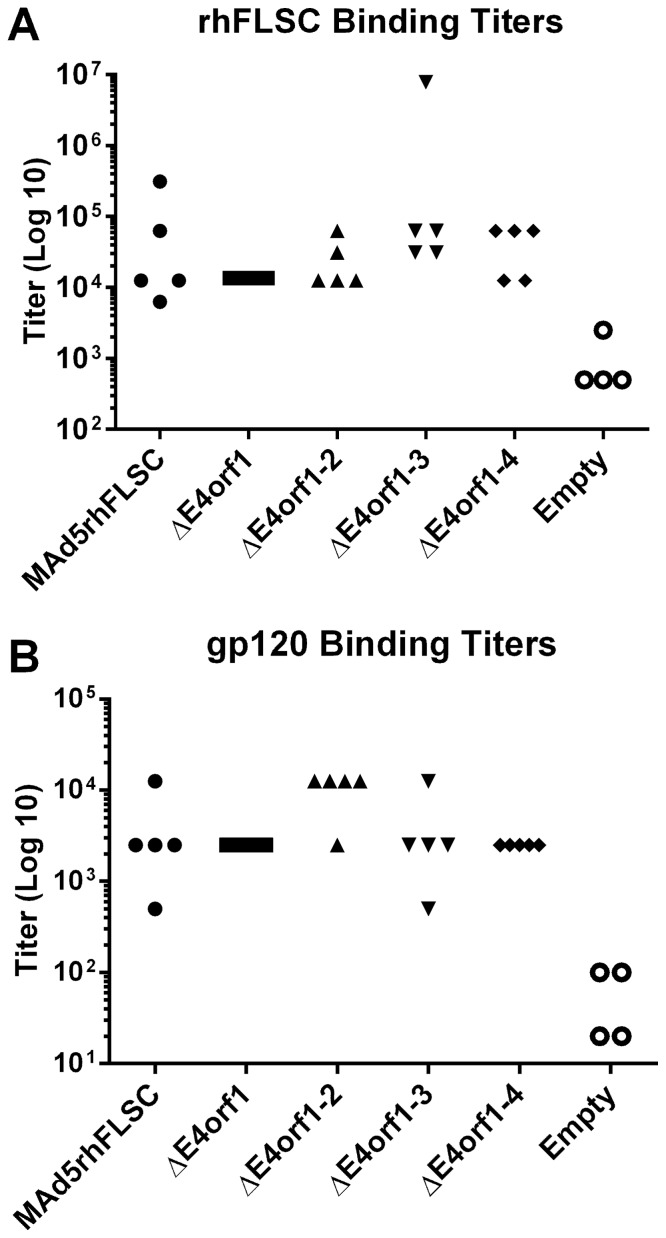
Deletion of *E4orf1* through *E4orf4* has little to no effect on antibody binding or specificity. (**A–B**) Sera collected from vaccinated mice were tested for specific binding to rhFLSC or HIV_BaL_gp120 proteins by ELISA. Log base 10 titers for each mouse were plotted and differences in comparison to titers obtained using sera from the control empty vector mice were analyzed. No differences were observed between the parent and the *E4*-deleted viruses (p>0.05). The binding titers of the combined vaccinated groups were significantly different from those of the empty vector group (p<0.002).

To determine the effects of the deletions on antibodies induced against the transgene, serum from each mouse was evaluated by ELISA. The mean rhFLSC and gp120 binding titers exhibited by sera from mice vaccinated with the parental virus or E4-deleted variants were significantly higher than those obtained for mice inoculated with the empty vector. Moreover, the values obtained for the group immunized with the parental vector were similar to those obtained from mice vaccinated with the *E4*-deleted variants (Figure 9A-B). Therefore viruses with deletions of *E4orf1* to *E4orf4* maintain transgene-specific antibody responses.

### Sera from mice vaccinated with Ad vectors deleted for *E4orf1* through *E4orf4* differentially recognize Ad antigens

**Figure 10:**
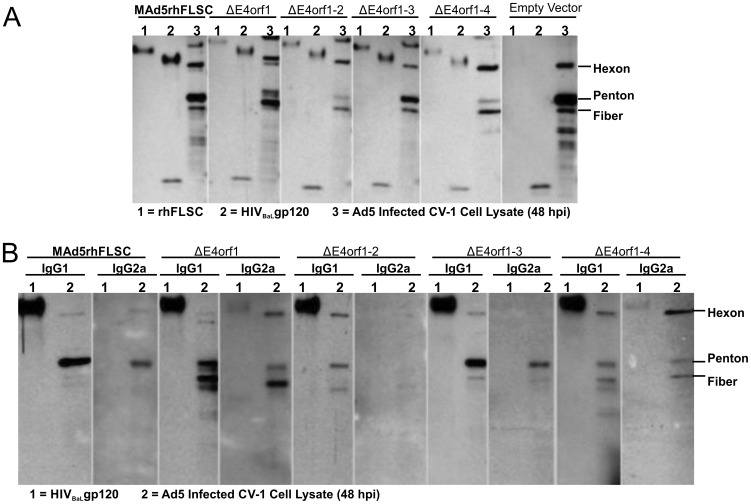
Sera from mice vaccinated with MAd5rhFLSC or the *E4*-deletion variants differentially recognize Ad antigens. (**A**) Equal amounts of recombinant rhFLSC protein, HIV_BaL_gp120 protein, or lysate from CV-1 cells collected 48 hpi were separated by SDS-PAGE, transferred to nitrocellulose, and exposed to pooled sera from mice vaccinated with the indicated viruses. The washed blots were then exposed to HRP conjugated anti-mouse IgG. (**B**) Similarly, blots of HIV_BaL_gp120 protein and lysate from CV-1 cells collected 48 hpi were incubated with pooled mouse sera and then with HRP conjugated anti-mouse IgG1 or to HRP conjugated anti-mouse IgG2a as indicated. Figures shown are representative of 2–5 experiments.

Serum samples within each group of immunized mice were pooled and the pools were examined for antigen recognition by western blot. All the pooled sera with the exception of that obtained from the non-transgene carrying empty vector group recognized both the rhFLSC and HIV_BaL_gp120 proteins (Figure 10A). Interestingly, while all the pooled sera from the vaccinated mice recognized the three most immunogenic Ad proteins (hexon, penton, and fiber), the intensity of those individual bands varied depending on the pooled sera used. Sera pooled from MAd5rFLSC, *ΔE4orf1–3*, and empty vector-vaccinated mice recognized the penton most strongly and fiber the least. By contrast, sera from Δ*E4orf1*, Δ*E4orf1–2* and Δ*E4orf1–4*-vaccinated mice recognized fiber more strongly than penton (Figure 10A).

In mice IgG2a is suggested to be the predominant Ig-subtype produced in response to viral infections [37]. However, in response to replication-defective Ad the specific Ig-subtype elicited may depend on the route of vaccination [38]. In mice immunized by replication-defective Ad through the intraperitoneal route, IgG2a against the hexon protein predominated. A more complex picture was seen following intravenous immunization where IgG2a initially predominated but gave way to the other Ig-subtypes over time [38]. To determine which Ig-subtype was produced in response to our vectors we analyzed the pooled sera from each group of virus-vaccinated mice by western blot. Unequivocally the transgene was predominantly recognized by IgG1 regardless of the presence or absence of *E4orf1* through *E4orf4* (Figure 10B). It was also weakly recognized by IgG2a antibodies but only in Δ*E4orf1*, and *ΔE4orf1–4* vaccinated mice. Interestingly, with the exception of mice vaccinated with the ΔE4orf1–2 virus that elicited predominantly IgG1 antibodies, all the other vaccinated mice produced both IgG1 and IgG2a antibodies against the viral proteins (Figure 10B). In mice vaccinated with the Δ*E4orf1* and *ΔE4orf1–4* viruses, hexon was recognized predominantly by the IgG2a sub-type. In the remaining vaccinated mice the Ad proteins were predominantly recognized by IgG1 subtype antibodies (Figure 10B). Independent of the Ig-subtype induced, the differential recognition of the Ad proteins, shown in Figure 10A, persisted. Thus our data suggest that sera from mice vaccinated with Ad vectors with deletions of *E4orf1* through *E4orf4* differentially recognize Ad antigens. These data indicate a possible role for the *E4* gene products in viral-host immune interactions that could impact vaccine applications. Further studies are required to explore this possibility.

## Discussion

Replicating Ad is currently being developed as a delivery vector for use in both HIV/AIDS and influenza vaccines, however its 3kb carrying capacity limits the size of the transgene that can be inserted. This size limitation undermines the usefulness of the replicating vector. One means of adding space in the vector is by deleting *E4orf1–4*. Others have created Ad with deletions of *E4orf1* through *E4orf4* [16], [17] however these vectors were not deleted in *E3* and did not contain a transgene. Therefore, effects of the *E4* deletions on transgene expression and immunogenicity could not be evaluated. To address this we created replicating, *E3*-deleted Ad type 5 host-range mutant (Ad5 hr) viruses bearing the rhFLSC transgene and systematically deleted the genes encoding early region 4 open reading frame 1 (*E4orf1*), *1–2*, *1–3* and *E4orf1–4*. The results of our study show that the deletions have little to no effect on virus host-cell interaction, transgene expression, T-cell immunogenicity, or transgene-specific antibody binding titers. That the transgene did not accumulate in the early phase of the virus replication cycle as would be expected for CMV and RSV driven transgenes suggests that here the transgene is regulated in a manner similar to Ad late proteins. Therefore it may be possible to create Ad vaccine vectors where the levels of transgene expression can be controlled. This could be of particular importance with regard to insertion of immune modulators whose expression levels might need to be tightly controlled.

Ad infected cells do not follow the canonical cell cycle progression but rather accumulate DNA content greater than G2/M cells (Figure 4). This has been reported for cells infected with wild type [39]–[41], and even *E1B55K*-deleted Ad [42]. That this occurs in cells as varied as cervical carcinoma-derived, glioblastoma-derived, and African green monkey kidney-derived lines infected with an *E3*-deleted vaccine vector, with or without further deletions in *E4* (Figure 4), suggests that it may be a feature common to the pathogenesis of replicating Ad. In animal and plant cells the accumulation of DNA content greater than that of G2/M cells is termed endoreduplication, and cyclin E may be required for it to occur. Recently it was suggested that the Ad E1B55K protein is required for enhancing cyclin E in infected cells [33]. This may be because in non-infected cells p53 induces the degradation of cyclin E [43] whereas in infected cells E1B55K in complex with E4orf6 programs the degradation of p53 accounting for the stabilization of cyclin E. We demonstrated the functionality of the E1B55K/E4orf6 complex by showing the disappearance of p53 in virus-infected cells over time (Figure 6). In synchronized infected HeLa cells we showed that cyclin E levels were the same at 12 and 24 hours post HU release in contrast to cells that were not infected (Figure 4). While it is tempting to suggest a role for cyclin E in Ad-induced endoreduplication, the fact that endoreduplication occurs in *E1B55K*-deleted virus-infected cells [42] where cyclin E should not be stabilized cast doubts on such a role. The precise role of cyclin E during Ad infection as well as the role of Ad-induced endoreduplication remain to be elucidated. The levels of Ad-induced endoreduplication can be regulated (Thomas et al., in preparation), which may lead to design of better replicating vaccine vectors.

Based on the similarities among the antibody binding titers within the groups of vaccinated mice (Figure 9) we pooled the sera. This did not influence the subsequent outcome that each of the recombinant viruses elicited predominantly IgG1 antibodies that recognized both rhFLSC and HIV_BaL_ gp120 proteins (Fig 10A, B), but may have obscured some mouse-to-mouse variation in recognition of different Ad proteins. Nevertheless, the suggestion of differential recognition of Ad structural components by sera of mice vaccinated with the parental or *E4*-deleted viruses is intriguing. Others have reported similar changes in humoral immune responses to the capsid component of Ad [38], supporting our results. However in this previous report they vaccinated with an E1 deleted vector, and as mentioned above E1A is required for efficient transactivation of the other early genes. Their vector also contained the E4 genes. Thus our study represents the first to look at the effects of deleting *E4orf1* through *E4orf4* on antibody responses to Ad structural components. A conclusive investigation using our recombinants would need to be in humans or a non-human primate model where these viruses replicate. In either model, it would be instructive to test the hypothesis that the Ad *E4* gene products either directly or indirectly influence antigen presentation in infected cells. This notion is not without support. In an *E1*-deleted virus the *E4* product was shown to protect infected cells from cytolysis nearly to the same degree as the *E3* region [44]. The E3–19K protein inhibits surface expression of MHC class I antigens by blocking the functions of TAP [45]-a cellular protein that binds cytosolic peptides and delivers then into the endoplasmic reticulum (ER). We do not expect the action of the *E4* proteins to be as direct as that of E3-19K. As mentioned, E4orf1, E4orf3, and E4orf4 have been reported to act on PI3 kinase and Rac1 [18], PML and Sumo [19], and PP2A [23] respectively. These cellular proteins all influence antigen presentation [46]–[50]. Consequently, lack of one or more of these *E4* gene products may result in an alteration that can impact humoral responses. These possibilities remain to be tested.

In this study the transgene was preferentially recognized by IgG1-subtype antibody regardless of *E4*-deletions, with weak recognition by IgG2a antibody only by sera of mice immunized with the *E4orf1* or *E4orf1–4* deletion vectors. We cannot rule out the possibility that the preferential recognition of the transgene by IgG1 may be a consequence of the nature of the transgene itself. In fact in other studies, mice similarly immunized with an *E3*-deleted replicating Ad5 hr-recombinant encoding the HIV_89.6P_ gp140 envelope and boosted with the Env protein elicited IgG2a antibodies against the transgene more strongly than IgG1 (Demberg et al., unpublished). The predominance of the IgG2a antibodies was attributed to the Ad5 hr-HIV_89.6P_ gp140 priming immunizations. Ig-subtypes against Ad proteins were not determined. Here we show that Ad-specific antigens were recognized by both IgG1 and IgG2a-subtype antibodies with the exception of the Δ*E4orf1–2* group, which was recognized predominantly by IgG1 antibodies (Figure 10B). Hence both the nature of the transgene and the vector backbone can influence the antibody subtype induced, which in turn has bearing on antibody effector function. Mouse IgG2a binds to all Fcγ receptors and therefore can mediate a spectrum of activities including phagocytosis, antibody-dependent cellular cytotoxicity (ADCC), production of cytokines, and clearance of immune complexes. In contrast, mouse IgG1 is more limited in effector function and binds with low affinity only to the inhibitory receptor, FcγRIIB, and the activatory receptor, FcγRIII [51]. Functionally, mouse IgG2a is similar to human IgG1, which also binds all FcγRs. As above, given that Ad5 hr recombinants do not replicate in mice, the antibody profile might be quite different from that elicited by a productive viral infection. Hence the antibody subtypes elicited by these vaccine vectors in preclinical studies in non-human primates, where replication occurs similarly to that of Ad vectors in humans, remains to be determined. In this study, although weakly reactive, mice vaccinated with either the ΔE4orf1 or the ΔE4orf1–4 vectors induced IgG2a antibodies against the transgene, and more prominently the same Ig-subtype against Ad hexon protein (Figure 10B). Hence, it is possible that by manipulating the E4 gene products one can create vectors that elicit antibodies with improved effector function.

To conclude, we have shown that deleting *E4orf1* to *E4orf4* in a transgene-bearing virus already deleted of the *E3* region does not impair the ability of the virus to replicate, alter its interaction with host cells, or weaken its immunogenicity. This study is the first to explore the effects of *E4* deletions on the expression and immunogenicity of a transgene inserted in a replication-competent Ad vaccine vector. Our results strongly suggest that *E4orf1* through *E4orf4* are dispensable in such a replicating vaccine vector. This is most likely because the 55kd product encoded by the Ad *E1B* gene, either by itself or in complex with the product of the *E4orf6* gene, functionally compensates for the other *E4* gene products [40]. The differential recognition of Ad antigens by sera from mice vaccinated with the individual *E4*-deleted viruses suggests that these gene products may harbor other functions that remain to be elucidated. Importantly, we have created vectors that can accommodate larger transgenes than the replication-competent Ad currently being used. These vectors should allow for greater flexibility in vaccine design either by expressing multiple transgenes under the control of varied promoters or immune modulators aimed at improving immunogenicity and overall efficacy.

## Materials and Methods

### Cell culture

All cell lines were obtained from the American Type Culture Collection. Cervical carcinoma-derived HeLa cells, human embryonic kidney-derived 293 cells, glioblastoma-derived U87 cells, and African green monkey kidney-derived CV-1 cells were maintained in Dulbecco's modified Eagle's medium supplemented with 10% fetal bovine serum. Cell culture media and supplements were obtained from Life Technologies (Gaithersburg, MD). Cells were maintained at 37°C in a humidified atmosphere with 5% CO2.

### Viruses

A codon optimized expression plasmid containing the rhFLSC [26] was obtained from Dr. Barbara Felber (NCI). The steps used in creating the shuttle plasmid were similar to those described previously in detail [52]. The modifications were only those specific to the rhFLSC transgene. Table S1 contains the list of primers used here. In brief, we removed the BamHI site from within the rhFLSC sequence as the presence of this site would interfere with downstream steps. The Ad5 tripartite leader (TPL) and Kozak sequences were inserted upstream of the rhFLSC coding sequence. The bovine growth hormone poly-A signal sequence (BGHpA) was inserted downstream of the rhFLSC sequence. The TPL-rhFLSC-pA expression cassette (∼2.6 kb) was excised from the expression plasmid and inserted into the XbaI site of the pBRAd5ΔE3 shuttle plasmid that contains the Ad5 sequence from 59.5 to 100 map units (mu) with a 78.8–85.7 mu deletion in the E3 region. PCR was used to ascertain the presence of the rhFLSC and the DNA was sequenced to confirm proper orientation of the TPL-rhFLSC-pA cassette. Thus the pBRAd5ΔE3 (TPL-rhFLSC-pA) shuttle plasmid (Figure 1A row 1) was made and verified.

To create pBRAd5ΔE3 (TPL-rhFLSC-pA) shuttle plasmids containing E4-deletions we performed two rounds of PCR using the primers listed in Table S1. The first round of PCR was used to insert the deletions and the second round with a 15 bp overhang was used to insert unique MCS within the E4 region. These sites compensate for the fact that there are few unique cloning sites within Ad5. We digested the pBRAd5ΔE3 (TPL-rhFLSC-pA) shuttle plasmids with PsiI and by gel extraction isolated the larger fragment. Using clontech ‘In-Fusion’ technology (Clontech Laboratories, Inc., California) we inserted the products of the second PCR into the PsiI digested pBRAd5ΔE3 (TPL-rhFLSC-pA) shuttle plasmids yielding the products illustrated in Figure 1A rows 2–4. PCR was used to ascertain the presence of both the rhFLSC and the appropriate E4 deletions. This was further confirmed by DNA sequencing.

The steps used in creating the Ad recombinants were also similar to those described previously [52]. In brief the pBRAd5ΔE3 (TPL-rhFLSC-pA) shuttle plasmids were digested with BamHI, separated on a 0.8% agarose gel and the larger fragments isolated, constituting the right-hand part of the virus. To obtain the left-hand part, DNA isolated from wild-type Ad5 hr was digested with SpeI and the larger fragment was isolated. The left- and right-hand fragments were co-transfected with lipofectamine 2000 (Invitrogen) into QBI 293 cells, incubated at 37°C and monitored for the presence of cytopathic effect (CPE). Viral DNA was isolated using a QIAamp DNA Blood Mini Kit (QIAGEN) and the resulting Ad5 hr-rhFLSC recombinant candidates were screened by PCR (Figure 1B, C). Expression of either gp120 or rhFLSC was evaluated by Western blotting (Figure 5). The parental virus, Mad5rhFLSC, and each of the E4-deleted recombinant viruses were further purified by three rounds of plaque purification. Aliquots of each pure viral stock were amplified on 293 cells and purified twice by cesium gradient centrifugation. The concentrations of the viral stocks were determined by optical density (OD) and plaque forming units (PFU) by plaque assays on 293 cells. The values along with the OD/PFU ratio are listed in Figure 1D.

### Cell cycle synchronization

Synchronously dividing cells were obtained by hydroxyurea block as described elsewhere [53]. In brief, asynchronously dividing cells were exposed to 2 mM hydroxyurea (Sigma, St. Louis, MO) for 24 hours. The hydroxyurea-containing medium was replaced either by normal growth medium or virus containing medium. The cells were then collected 2, 4, 8, 12, and 24 hours post infection (hpi). The fraction of cells in each phase of the cell cycle was determined using propidium iodide staining and DNA analysis by flow cytometry.

### Virus yield

Viral yield was determined by plaque assay with 293 cells as described previously [18].

### Gel electrophoresis and Western Blot

Cells infected at a MOI of 5–50 were lysed directly in 1X protein sample buffer (1X SDS Gel Loading Dye, 10% BME). Equal amounts of samples of were run on 4–20% SDS-polyacrylamide gels (Life Technologies, Gaithersburg, MD). Proteins were transferred to nitrocellulose membranes using the iBlot Western Blot System (Life Technologies, Gaithersburg, MD). Blots were blocked in PBS-0.02% Tween 20, containing 20% milk, for 2 hours and thereafter exposed to a 10% milk buffer containing one of the following primary antibodies at 4° overnight or for 2 hours at room temperature: anti-Ad type 5 E1A, anti-cyclin E, (BD Biosciences, San Jose, CA); anti-actin (Sigma-Aldrich, St. Louis, MO); anti-p53 (Thermo Scientific, Rockford, IL); anti-HIV-1 gp120 (Meridian Life Sciences, Memphis, TN); anti-hCD4 (R&D Systems, Minneapolis, MN); and anti-Ad type 5 (Abnova, Walnut, CA). Subsequently, blots were washed and exposed to an HRP conjugated secondary antibody, either anti-mouse IgG, anti-human IgG, anti-rabbit IgG, anti-goat IgG (KPL, Gaithersburg, MD) or anti-mouse IgG1 or IgG2a (BD Biosciences) as dictated by the primary isotype. Chemiluminescent detection was performed using SuperSignal West Pico Chemiluminescent Substrate (Thermo Scientific) or LumiGLO Chemiluminescent Substrate System (KPL).

### Cell cycle analysis

DNA cell cycle analysis was performed by flow cytometry as described elsewhere [54]. Isolated cells were washed, fixed in 70% ethanol and thereafter exposed to an RNase solution containing propidium iodide. The PI stained cells were interrogated using a FACSCalibur and CellQuest Pro (BD Biosciences, San Jose, CA). The percentage of cells in each cell cycle phase was obtained and plotted in Flow Jo (Tree-Star Inc.).

### Animals and vaccination

Six- to eight-week-old female BALB/c mice were housed and maintained in a pathogen-free environment according to the standards of the American Association for Accreditation of Laboratory Animal Care at Advanced BioScience Laboratories, Inc. (ABL, Rockville, MD). The animal protocol was reviewed and approved by the Animal Care and Use Committee prior to implementation. Mice were inoculated intraperitoneally with 5.0×10^8^ PFU per dose according to the immunization schedule outlined in Figure 7A. At 6 weeks post-immunization spleens were collected and single-cell suspensions were obtained by passing them through a 70 µm nylon cell strainer with the help of a syringe plunger. The erythrocytes were lysed and the splenocytes washed and stored in freezing media (10% DMSO plus 90% FBS) in liquid nitrogen. At week 6 blood samples were also collected without anticoagulant. The clotted blood samples were centrifuged and sera were stored at −70°C until they were analyzed.

### Intracellular cytokine staining

Splenocytes (2×10^6^) were stimulated for 6 h at 37°C in the presence of HIV_BaL_gp120 or Ad5 fiber peptides (15-mers with an 11 amino acid overlap; ABL) at a 1 μg/ml final concentration of each in the presence of anti-CD28 (1 μg/ml) and brefeldin A (10 μg/ml). For each assay day, an unstimulated control and a positive control (Staphylococcus enterotoxin B) were included in the experiment. Cells were washed twice with PBS, resuspended in 95 μl of PBS plus 5 μl of AquaBlue viability dye (a 1∶50 dilution of a dimethyl sulfoxide stock; Invitrogen) and incubated at room temperature (RT) for 10 min. Cells were washed once with PBS and surface stained for 30 min at RT with CD4-PE-Cy5, CD8-APC, CD44-PE-Cy7, and CD62L-APC-Cy7 antibodies (BD PharMingen), at concentrations determined by dilution of stock or according to the manufacturer's instructions. Cells were then washed with PBS containing 2% fetal bovine serum (FBS), fixed with Cytofix/Cytoperm solution (BD Biosciences), permeabilized with 1× Perm/Wash, and incubated for 30 min at 4°C in the dark with CD3-PE, gamma interferon (IFN-γ)-Alexa 700, tumor necrosis factor alpha (TNF-α)-FITC, and interleukin-2 (IL-2)–PerCP-Cy5.5 antibodies (BD PharMingen). Cells were washed once with 1× Perm/Wash and once with PBS containing 2% FBS and resuspended in 1% paraformaldehyde in PBS. Approximately 500,000 lymphocytes were acquired for analysis using an LSRII Flow Cytometer. A singlet, followed by live/dead and then lymphocytic gates, were first applied. CD3^+^ T cells were divided into CD4^+^ and CD8^+^ populations, and each population was further subdivided into CD62L^hi^CD44^hi^ CM and CD62L^lo^CD44^hi^ EM cells. The percentage of cytokine-secreting cells in each memory cell subset was determined following subtraction of the values obtained with non-stimulated samples. Data were analyzed using FlowJo version 9.5.2 (Tree-Star Inc.).

### Antibody analyses

Antibody binding titers were assayed by enzyme-linked immunosorbent assay (ELISA). Ninety-six well plates were coated with 100 ng per well of either HIV_BaL_gp120 (ABL) or rhFLSC (Profectus BioSciences, Inc., Baltimore, MD). The plates were exposed to 1% BSA blocking solution (KPL) for 2 hours at room temperature. The serum samples were serially diluted and applied in duplicate to the 96-well plates and incubated at 4°C overnight. The next morning the plates were washed with PBS–Tween, exposed to peroxidase-conjugated goat anti-mouse IgG (H+L), and thereafter incubated for another hour. After washing the plates were developed with TMB (3, 3′, 5, 5′-tetramethylbenzidine) peroxidase substrate solution. The reaction was stopped by adding 1M H_3_PO_4_ and the plates were read at 450 nm within 30 min. An OD threshold value of 0.3 was used to determine end-point titer.

### Statistical analysis

Initial statistics were obtained using Prism v6.0 (GraphPad Software Inc.) and further confirmed using SAS/STAT software (SAS Institute Inc., Cary, NC). Differences between viral progeny production of the parental and *E4*-deleted viruses were analyzed by one-way ANOVA. Differences between groups in total cytokine positive cells were analyzed by repeated measures analysis of variance following arc-sine transformation of the values. Differences in antibody titers between groups were analyzed by the Kruskal-Wallis test. P-values ≤0.05 were deemed significant.

## Supporting Information

Table S1 **PCR Primers used in the creation of rhFLSC Ad5 hr variants deleted of E4orf1, 1–2, 1–3, and 1–4.**
(DOC)
