# Supplementary material for: Effects of the Deletion of Early Region 4 (E4) Open Reading Frame 1 (orf1), orf1-2, orf1-3 and orf1-4 on Virus-Host Cell Interaction, Transgene Expression, and Immunogenicity of Replicating Adenovirus HIV Vaccine Vectors
Source: PLoS One. 2013 Oct 15;8(10):e76344. doi: 10.1371/journal.pone.0076344 (PMC3797075; doi:10.1371/journal.pone.0076344)
Supplement: Table S1 — PCR Primers used in the creation of rhFLSC Ad5 hr variants deleted of E4orf1, 1–2, 1–3, and 1–4. (DOC) [file pone.0076344.s001.doc]

**Table S1.** **PCR Primers used in the creation of rhFLSC Ad5hr variants deleted of E4orf1, 1-2, 1-3, and 1-4.**

| **Name** | **Polarity** | **Sequence (5'**🡒**3')** |
| --- | --- | --- |
| rhFLSC.BamHI.KO.S | Forward | GTG CAG CGC GAG AAG ACC GGt TCC TCT GGT G |
| rhFLSCopt.BamHI.AS | Reverse | CCG GTC TTC TCG CGC TGC ACG ACC CGG CGC |
| BGHpA.EcoRI.F | Forward | TAG AAT TCT GTG CCT TCT AGT TGC CAG CC |
| BGHpA.AvrII.R | Reverse | TAC CTA GGC CAT AGA GCC CAC CGC ATC CCC AGC ATG ATG CCT GCT ATT GT |
| IF.Ad5TPL.AvrII.rhFLSC.F | Forward | AAG AGG TAC CGT CGA CCT AGG CAC TCT CTT CCG CAT CGC TG |
| IF.AD5TPL.NcoI.rhFLSC.R | Reverse | TGC AGC GAC CCC ATG GTG GCC CTT GCG ACT GTG ACT GGT TAG ACG CCT TT |
| rhFLSCopt.Seq#2 | Forward | GGC GAG ATG AAG AAC TGC AG |
| rhFLSCopt-P2 | Reverse | ATC ACG ACC TCC TCC TCA GC |
| Product length: 365bp |  |  |
| Ad5E3-P1 | Forward | TACGAGAGAACCTCTCCGAG |
| Ad5E3-P2 | Reverse | ACAGGCTGGCTCCTTAAAAT |
| E4orf1-4 deletion primers | | |
| E4orf1, 1-2, 1-3, 1-4 | Forward | ACAGGGCCCTCTTTTTATAA |
| E4orf1 | Reverse | ATGTTTTTTTGTTATTTTATTT |
| E4orf1 | MCS | CGTCTTATGCTTATAATTGGGCCGGCCTGTATGCATAATGTTTTTTTGTTATTTTATTT |
| E4orf1-2 | Reverse | AATCATGATTCGCTGCTTGA |
| E4orf1-2 | MCS | CGTCTTATGCTTATAATTGGGCCGGCCTGTATGCATAATCATGATTCGCTGCTTGA |
| E4orf1-3 | Reverse | CATGGTTCTTCCAGCTCTTC |
| E4orf1-3 | MCS | CGTCTTATGCTTATAATTGGGCCGGCCTGTATGCAT CATGGTTCTTCCAGCTCTTC |
| E4orf1-4 | Reverse | ATATGACTACGTCCGGCGTT |
| E4orf1-4 | MCS | CGTCTTATGCTTATAATTGGGCCGGCCTGTATGCAT ATATGACTACGTCCGGCGTT |
|  |  | Psil                   Fsel                   Nsil |

Enzyme recognition sites are underlined. Mutated nucleotide is indicated by a lower case letter.
